# Exploring the Effects of High Protein and High Inulin Composite Shrimp Surimi Gels on Constipated Mice by Modulating Gastrointestinal Function and Gut Microbiota

**DOI:** 10.3390/foods15010059

**Published:** 2025-12-24

**Authors:** Yuting Tan, Peizi Sun, Chen Tao, Yajie Qin, Huimin Liu, Dongmei Li

**Affiliations:** 1 National Engineering Research Center of Seafood, School of Food Science and Technology, Dalian Polytechnic University, Dalian 116034, China; tanyuting201130@163.com (Y.T.); sunpz1997@163.com (P.S.); taochen0122@163.com (C.T.); m13504118437@163.com (Y.Q.); mtst2415@163.com (H.L.); 2 Collaborative Innovation Center of Seafood Deep Processing, Dalian Polytechnic University, Dalian 116034, China; 3 State Key Laboratory of Marine Food Processing & Safety Control, Dalian Polytechnic University, Dalian 116034, China

**Keywords:** inulin, Antarctic krill, constipation, gut microbiota

## Abstract

This study aimed to develop a dietary fiber-rich Antarctic krill composite shrimp surimi gel (AKSG) and to investigate the improvement effects of high protein (HP), high protein and dietary fiber (HPDF), and high dietary fiber (HDF) diet interventions on constipation behaviors and gut microbiota of mice. The results showed that the HPDF group significantly improved defecation in constipated mice, enhanced gastrointestinal peristalsis, and exhibited the most obvious effect on improving the colonic structure. The gut microbial analysis showed that the HPDF group increased the relative abundance of beneficial bacteria and improved the intestinal microbial environment of constipated mice. In addition, all groups effectively regulated the secretion of intestinal neurotransmitters. Inulin significantly increased the fecal water content by binding to water molecules, thus softening feces. Meanwhile, the addition of an appropriate amount of protein could further absorb water in the intestinal tract and relieve constipation. In conclusion, dietary fiber-rich AKSG might be a promising nutritious functional food for constipation relief.

## 1. Introduction

Constipation is one of the most common functional gastrointestinal disorders [1]. It has a global average prevalence of about 15%, affecting people of all ages [2]. Constipation causes pain and leads to numerous complications, such as cardiovascular disease, bowel perforation, colon cancer, inflammation, rectal prolapse, piles, and hemorrhoids [3,4]. It is characterized mainly by reduced intestinal transport capacity, prolonged colonic transit time, and retention of colonic contents, which are the manifestations of insufficient intestinal motility [5]. Constipation has a complex etiology with numerous factors, such as diet, lifestyle, and gut microbiota, playing important roles in its development [6]. Some studies have shown that soluble fiber and trace minerals, among others, can reduce the risk of constipation [7]. Through its hydration properties and fermentability, dietary fiber promotes intestinal peristalsis, improves gut microbiota, and increases fecal bulk and is widely regarded as an effective means of relieving constipation [8]. Dietary fibers can increase stool volume and promote defecation; moreover, they are fermented by gut microbes to produce short-chain fatty acids, which regulate the release of intestinal neurotransmitters and improve intestinal motility [9]. Zhang et al. [10] investigated whether chitosan oligosaccharides (COSs) alleviate constipation through gut microbiota modulation. Their results indicated that COSs significantly improved intestinal motility, attenuated inflammatory responses, normalized water and electrolyte metabolism, and preserved intestinal barrier integrity. With increasing global health awareness, the use of effective and safe dietary interventions to prevent and relieve constipation has become a focus of public and scientific research [11].

Inulin is a natural water-soluble dietary fiber and an abundant polysaccharide found in nature in a variety of plants such as chicory and garlic [12]. Inulin is also a high-quality prebiotic that can improve the gut microbiota structure, promote the proliferation of probiotics, regulate blood glucose levels, and reduce the risk of colon cancer and other diseases [13]. Due to its unique chemical and prebiotic properties, inulin has a wide range of applications in functional foods [14]. Cao et al. [15] loaded a probiotic inulin hydrogel with polypyrrole nano-enzymes and antifibrotic drug pirfenidone and investigated their simultaneous improvement effects on inflammatory bowel disease (IBD) and its fibrotic complications. The inulin complex enhanced intestinal epithelial barrier repair, and inhibited intestinal fibrosis by modulating the gut microbiota and inhibiting fibroblast proliferation. Lan et al. [3] evaluated the effects of inulin (INU) and isomalto-oligosaccharide (IMO) in a rat constipation model induced by diphenoxylate. The results demonstrated that both prebiotics increased colonic levels of motilin (MTL), substance P (SP), and short-chain fatty acids (SCFAs), while decreasing vasoactive intestinal peptide (VIP) and calcitonin gene-related peptide (CGRP). Furthermore, prebiotic supplementation increased *Lactobacillus* abundance and altered the overall gut microbiota structure. Beyond these physiological effects, the technological utility of inulin in food systems is equally important. The molecular structure of inulin is rich in hydroxyl groups, which form hydrogen bonds with water molecules, thus forming a stable gel structure. Therefore, inulin has good water solubility and rheological properties and is usually used as an emulsifier or fat substitute in meat products [16]. Chen et al. [17] prepared a dietary fiber-rich fish-derived protein gel using an inulin–Konjac glucan (KGM) complex. They showed that the 8% inulin–KGM complex could reduce the deformation resistance and hardness, and improve the gel properties of the myofibrillar protein (MP) gel.

The Antarctic krill is an arthropod. It has an estimated biomass of about 379 million tons and is a representative biological resource in Antarctic waters. It is an extremely important link in the food chain of Antarctic waters, playing a key role in coordinating the balance of the ecosystem [18]. As a high-quality protein source, Antarctic krill contains eight essential amino acids, with the total amount accounting for more than 20% of the total protein content [19]. The amount of essential amino acids provided by Antarctic krill proteins meets what is required by healthy adults, that is similar to milk protein contents [20]. It not only meets the demand for protein, vitamins, and minerals [21], but also helps in preventing cardiovascular diseases and improving overall health. Xu et al. [22] investigated how long-term high-protein (HP) and konjac glucomannan (KGM) diets affect metabolic health. They found that an HP diet improved lipid homeostasis by downregulating the fatty acid synthase gene, with no adverse effects. Notably, the HP + KGM combination offered further benefits by downregulating hepatic genes and reducing colonic TNF-α expression, thereby enhancing both glucose and lipid metabolism. However, due to its rich enzyme system, the autolysis during its fishing can lead to the deterioration in protein quality [23]. This makes the development of Antarctic krill products more difficult. Shrimp gel is characterized by its high protein content, low fat content, and richness in minerals and other nutrients, serving as an ideal high-protein food matrix [24]. Chen et al. [25] investigated the effect of κ-carrageenan on the physicochemical and structural properties of ready-to-eat (RTE) Antarctic krill surimi gel. Their findings indicate that incorporating 4% (*w*/*w*) κ-carrageenan significantly reduced cooking loss, enhanced water-holding capacity and textural quality, and promoted the formation of hydrophobic interactions and disulfide bonds. However, SDS-PAGE analysis confirmed that these changes occurred without altering the primary molecular composition of the proteins. Currently, despite numerous studies on Antarctic krill protein in recent years, there are fewer gel products based on Antarctic krill protein. In-depth studies focusing on the development and utilization as well as its structure and function should be performed.

Swallowing is a complex phenomenon, involving multiple reflex activities, which deliver food through the mouth and esophagus to the stomach; a problem with any of these can lead to swallowing disorders [26]. People with dysphagia usually eat small amounts of food, which can lead to inadequate intake of nutrients, such as dietary fiber, minerals, and vitamins; this can increase the risk of malnutrition, constipation, and pneumonia [27]. Moreover, insufficient fiber and water intake can result in insufficient stool volume to effectively stimulate defecation, which is one of the causes of chronic constipation. Currently, dysphagia foods are confusingly categorized, single-structured, and low-tech, and the research mainly focuses on texture improvement, while other aspects, such as nutrition and function, are ignored. Most of the dysphagia foods that meet texture requirements lack nutritional intake. Therefore, people with dysphagia as well as elderly people have higher nutritional requirements, and the development of nutritional functional foods rich in dietary fiber and easy to chew is important to improve the health status of people with dysphagia [28]. In this study, inulin and sodium alginate (SA) were added to increase the contents of dietary fiber in Antarctic krill composite shrimp surimi gels (AKSGs), which increased the nutritional value of the product and conformed to the swallowing requirements of people with dysphagia. Moreover, this study investigated the physiological and functional properties of the product in terms of its effect on relieving constipation.

## 2. Materials and Methods

### 2.1. Materials and Chemicals

Inulin powder, egg white powder, acetylated distarch phosphate and lysine were obtained from Henan Wanbang Industrial Co., Ltd. (Zhengzhou China). Sodium alginate powder was obtained from Qingdao Mingyue Seaweed Group Co., Ltd. (Qingdao, China). Antarctic krill and *Litopenaeus vannamei* were obtained from Dalian Liaoyu Yuanyang Food Co., Ltd. (Dalian, China). Loperamide hydrochloride was obtained from Janssen Pharmaceutical Company (Xi’an, China).

### 2.2. Preparation of Composite Shrimp Surimi Gels

AKSGs were prepared following a modified protocol derived from previously reported methods [29]. The Antarctic krill and *Litopenaeus vannamei* were weighed at a 2:3 mass ratio and then ground using a meat grinder for 3 min. Ice water (15%, *w*/*w*) and NaCl (1%, *w*/*w*) were added to the mixture and mixed again for 3 min. Soybean oil and monosodium glutamate were added each at 0.5% (*w*/*w*), while other ingredients were added in five quantities each to make gels of five different compositions as follows: 2%, 3%, 4%, 5%, and 6% (*w*/*w*) inulin; 1%, 2%, 3%, 4%, and 5% (*w*/*w*) egg white powder (EWP); 1%, 2%, 3%, 4%, and 5% (*w*/*w*) acetylated distarch phosphate (ADSP); 0.3%, 0.5%, 0.7%, 0.9%, and 1.2% (*w*/*w*) sodium alginate (SA); and 0.2%, 0.3%, 0.4%, 0.5%, and 0.6% (*w*/*w*) lysine (Lys). The mixture was then continuously mixed for an additional 3 min. The prepared AKSGs were vacuum packed in a cooking bag after heating and sterilized in stages: 100 °C for 10 min and 115 °C for 15 min.

Except where otherwise described in the sample preparation, inulin, EWP, and ADSP were 3%, SA was 0.5%, and Lys was 0.4%. The specific formulation is shown in Table 1. The hardness was performed to obtain the optimal single-factor conditions, which were then used for response surface analysis based on Box–Behnken design principles.

### 2.3. Texture Profile Analysis (TPA)

The textural properties of the composite gels were determined using a TA-XT plus texture analyzer (Stable Micro Systems Ltd., Godalming, UK). A P/50 probe was used with the test speeds set to 1 mm/s [30].

### 2.4. IDDSI Test

Samples were classified into 8 classes based on thickening (0–7). Based on the preliminary tests, the composite gel was categorized as class 5 and class 6 and had to pass the spoon tilt test, fork drip test, and fork and spoon pressure test recommended by the IDDSI framework [31].

### 2.5. Animal Experiment

Healthy male BALB/c mice (aged six weeks) were purchased from the Hubei Provincial Center for Disease Control and Prevention (Wuhan, China). All the mice were housed under a controlled environment (room temperature of 23 ± 2 °C with 12 h/12 h light/dark cycles) and provided with water and a normal chow dietad libitum. All the animal experiments were approved by the Ethical Experimentation Committee of Dalian Polytechnic University and the National Act on the Use of Experimental Animals (China).

After one week of acclimatization, the mice were randomly divided into five groups (*n* = 12): control, model group, high protein group (HP), high protein and dietary fiber group (HPDF), and high dietary fiber group (HDF). The experimental diets were prepared as follows: freeze-dried samples from two AKSG groups were added to replace 30% of the standard chow, yielding the HP diet and HPDF diet. An additional HDF group was prepared by supplementing the diet with SA and inulin at 6.5% substitution. The specific formulation is shown in Table 2. No additional exercise intervention was administered during the experimental stage, and daily water intake was standardized across all cages. The control group mice were fed standard chow without any treatment. Constipation was induced in the rest of the groups by the oral gavage administration of 10 mg/kg loperamide hydrochloride once a day for 7 days to establish the constipation model. On day 4 after loperamide hydrochloride induction, the regular feed was replaced with the corresponding customized feed and fed for ten consecutive days. The general physiological status and fecal condition of the mice were recorded daily during the test period.

### 2.6. Fecal Indicators

#### 2.6.1. Determination of Fecal Water Content

The mice were placed in metabolic cage boxes, and their feces were collected for 4 h. The wet weight W_1_ was measured. Then, the feces of all groups were placed in a 100 °C high-temperature oven and dried continuously for 2–4 h. Then, the dry weight W_2_ was measured, and the fecal water content was calculated [32].

$$Water\;content\;of\;fecal\left(\%\right)=\left(W_{1}-W_{2}\right)/W_{1}\times 100\%$$

#### 2.6.2. Determination of the Time of First Black Feces and Number of Fecal Pellets

One hour after the end of the gavage on day 14 of the experiment, all mice were gavage-administered with 0.2 mL of ink and placed individually in the metabolic cage. The time between gavage and the first black fecal pellet was calculated as the time of the first black fecal pellet. The number of black fecal pellets excreted by the mice over a period of 6 h was recorded.

### 2.7. Indicators of Gastrointestinal Motility

#### 2.7.1. Small Intestine Advancement Rate

After 1 h of the gavage of loperamide hydrochloride, mice were gavage-administered with 0.2 mL of ink each and sacrificed with cervical dislocation after 30 min. Their small intestines were removed intact and straightened, and the ink advancement length measured [33].

Small intestine advancement rate(%)=Ink advance length/Total length of small intestine×100%

#### 2.7.2. Gastric Emptying Rate

For the small intestine motility experiment, the whole stomach of mice was cut off and weighed. The stomach was cut along the large, curved surface. The stomach contents were washed with saline and blotted dry on filter paper, followed by weighing the net weight of the stomach [33].

$$Gastric\;emptying\;rate\left(\%\right)=\left[1-\left(\frac{Full\;stomach\;weight-Net\;stomach\;weight}{Full\;stomach\;weight}\right)\right]\times 100\%$$

### 2.8. Intestinal Fluorescence Imaging

One hour after the end of the gavage on day 14 of the experiment, mice were euthanized. The intact intestinal tissues were extracted, and their fluorescence imaging was performed using a small animal fluorescence imager to detect the distribution and content of chlorophyll. The extracted intestinal tissues were laid flat on a black background plate to avoid folding and overlapping. The excitation wavelength was set at 488 nm with the emission wavelength at 650 nm [34].

### 2.9. Histopathological Examination

The colon tissues were fixed, embedded, sectioned, and then subjected to hematoxylin and eosin (H&E) staining, AB-PAS staining, and immunofluorescent staining. Finally, the sections were imaged by light microscopy and quantified using ImageJ 1.54g software [35].

### 2.10. Enteric Neurotransmitter Assay

The mice serum samples were collected, and the contents of motilin (MTL), substance P (SP), somatostatin (SS), and vasoactive intestinal peptide (VIP) were determined using the ELISA kits (Shanghai Enzyme Link Biotechnology Co., Ltd., Shanghai, China) following the manufacturer’s instructions. The detection wavelength was 450 nm [35].

### 2.11. 16S rRNA Gene Sequencing

*16S rRNA* gene sequencing was performed as described by Logue et al. [36] with partial modifications. Briefly, the mice’s fecal samples were weighed (400 mg), and total DNA was extracted using a fecal genomic DNA extraction kit with the magnetic bead method. The extracted DNA was tested for purity, concentration, and integrity. The V3–V4 variable region of the 16S rRNA gene was amplified using PCR. The PCR amplicons were purified and quantified, and the qualified gradient dilution of each up-sequencing library was mixed in an appropriate ratio according to the desired sequencing volume and denatured with NaOH to single strands for up-sequencing. Subsequently, the 2 × 250 bp paired-end sequencing was performed. The bipartite sequencing data was split based on the barcode information, and the splice and barcode sequences were removed. Finally, data splicing and filtering were performed.

### 2.12. Statistical Analysis

Statistical analysis was performed using IBM SPSS 27. Significant differences, defined at *p* < 0.05, in the data were analyzed using ANOVA. The data of each experiment were repeated at least three times, and expressed as mean ± standard deviation (SD). The response surface test was conducted using Design-Expert 13. Gene sequencing data were processed on the Omic Studio platform. Graphs were created using Origin 2021 and GraphPad Prism 9.5.0 software. The analysis of acquired images from histopathological imaging was performed using Case Viewer and ImageJ 1.54g software.

## 3. Results and Discussion

### 3.1. Single Factor Test

**Figure 1:**
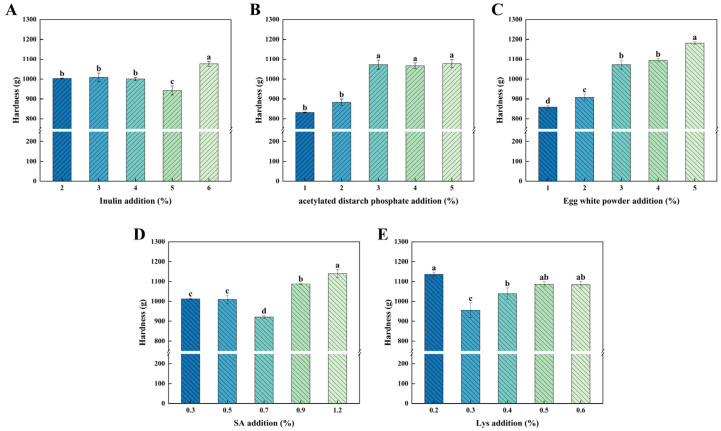
Hardness of AKSG with different addition amounts of inulin (**A**), ADSP (**B**), EWP (**C**), SA (**D**), and Lys (**E**). Values of different groups with different lower case letters (a–d) are significantly different at *p* < 0.05.

Processing methods and functional additives are the main factors affecting the gelation properties of minced shrimp. Suitable and appropriate amounts of exogenous additives can improve the gelation performance as well as the functional properties of minced shrimp gels [37]. Exogenous additives, which are used to improve gel properties, mainly focus on dietary fibers, starchy polysaccharides, proteins, oils and fats, and some small molecules [38]. As shown in Figure 1A, the increase in inulin addition first decreased the hardness course of the AKSG and then increased it, with the lowest hardness (942.84 g) at 5% inulin addition. ADSP has a lower pasting temperature and better anti-aging properties. With the increase in ADSP, the hardness increased (Figure 1B). During the heating process, starch forms a homogeneous gelatinous 3D network structure by interacting with water and proteins, which in turn increases the hardness. Increasing EWP concentration directly raised hardness (*p* < 0.05, Figure 1C), with a minimum of 858.97 at 1%. SA has hydrocolloid properties and can enhance the functional properties of food products. The hardness value of the AKSG was lowest at 0.7% SA addition (920.99 g) and increased significantly (*p* < 0.05) with the increase in SA concentration (Figure 1D). Therefore, 0.7% SA was considered to be the optimal addition amount for AKSG. With the increase in the amount of Lys added, the hardness showed a first increasing and then decreasing trend with the lowest hardness value when the amount of addition reached 0.3% (Figure 1D). Therefore, 0.3% Lys addition was determined to be optimal. In summary, the optimal amount of exogenous additives in the AKSG was 5% inulin, 1% ADSP, 1% EWP, 0.7% SA, and 0.3% Lys.

### 3.2. Response Surface Optimization Experiment and IDDSI Test

Based on the response surface test factors and levels (Table S1), the experimental protocol and results are summarized in Supplementary Table S2. Using multivariate fitted regression resulted in the following fitted equations and Table S3:

Y = 780.30 − 48.01*A* − 8.13*B* − 20.39*C* + 29.80*AB* − 10.58*AC* + 14.37*BC* + 29.48*A*^2^ + 69.31*B*^2^ − 22.92*C*^2^

**Figure 2:**
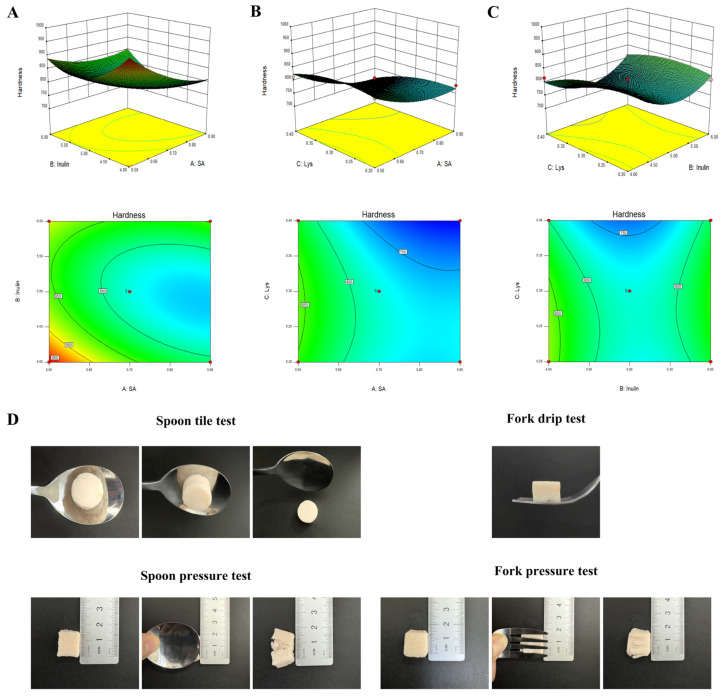
Surface and contour plots of inulin and SA (**A**), SA and Lys (**B**), inulin and Lys (**C**), and the IDDSI test (**D**).

As shown in the 3D response surface and 2D contour plots among SA, inulin, and Lys concentration (Figure 2), the interaction between inulin and SA was the highest. The hardness index of AKSG showed a steep arc shape with the contour plot tending to be elliptical, indicating that the hardness first decreased and then increased with the increase in the addition of SA and inulin. This might be due to their strong solubility and more hydrophilic groups, which can form hydrogen bonds with water molecules and then form a dense gel network structure during the heating process [29]. AKSG exhibited the lowest hardness with the following exogenous additives: 0.9 g SA, 5.49 g inulin, and 0.38 g Lys. According to the operability, with the addition of 0.9 g SA, 5.5 g inulin, and 0.4 g Lys, the hardness reached 668.275 g, which was close to the hardness predicted by the model.

As shown in Figure 2D, the spoon tilt test results showed that the samples had smooth surfaces with no residue, which met the requirements for difficult-to-swallow foods in terms of adhesion and cohesion [39]. In the fork drip test, the sample did not leak between the fork tines. The results of the pressure test showed that the AKSG could be easily flattened and crushed. Upon removal of the pressure, the shape did not recover with no agglomerates or visible particles [40]. The results suggested that the high dietary fiber composite shrimp surimi gel could be identified as level 6 in the IDDSI framework.

### 3.3. Effect of Dietary Intervention on the Body Weight and Fecal Indicators of Constipated Mice

**Figure 3:**
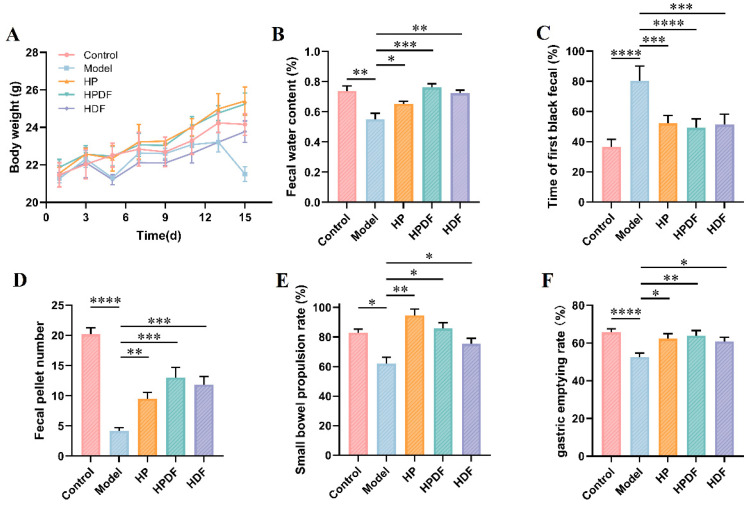
Effect of dietary intervention on the body weight, fecal indicators, and gastrointestinal motility indicators of constipated mice. (**A**) Recording the body weight of mice. (**B**) Fecal water content. (**C**) Time of first black fecal pellet. (**D**) Fecal pellet number. (**E**) Small bowel propulsion rate. (**F**) Gastric emptying rate. Data were represented as mean ± SD (*n* = 6). * *p* < 0.05, ** *p* < 0.01, *** *p* < 0.001, **** *p* < 0.0001. HP: high protein diet; HPDF: high protein and dietary fiber diet; HDF: high dietary fiber diet.

The effects of changes in different dietary interventions on the body weight of mice are shown in Figure 3A. The body weights of all groups increased compared with those of the model group, especially the protein-containing HF and HPDF groups, which showed a significant trend of increasing body weights after the gradual change in diets. The fecal water content of mice was significantly lower in the model group compared to the control group (*p* < 0.01, Figure 3B). The dietary intervention of constipated mice significantly increased the fecal water content of mice, especially in the HPDF (*p* < 0.001) and HDF (*p* < 0.01) groups, where fecal water content reached 76.32% and 72.39%, respectively. The fecal water content was higher in the HPDF group than in the control group; however, the difference was insignificant. Dietary fiber significantly increased the water content of feces by binding to water molecules, thus softening feces [41,42]. Moreover, the amino acids produced by protein digestion can increase the osmotic pressure of the small intestine, absorb water into the intestinal tract, and further soften feces [43]. The time to discharge the first black fecal pellet of mice in the in the HPDF (*p* < 0.0001), HP, and HDF (*p* < 0.001) groups shortened significantly compared to the model group at 49.17 min, 52.17 min, and 51.33 min (Figure 3C). The difference in the time to discharge of the first black stool in mice in the HPDF group was the shortest compared to that in the control group. In conclusion, HPDF significantly improved the phenomena of constipation-induced defecation volume, decreased fecal water content, and prolonged defecation time in mice, with effects better than those of the HP and HDF groups.

Reduced defecation is an important and one of the most visual manifestations of constipation [44]. Fecal indicators were evaluated in constipated mice by counting the 6 h defecation pellets. As shown in Figure 3D, the fecal number in the HP (*p* < 0.01), HPDF, and HDF (*p* < 0.001) groups was increased by 127.82%, 211.75%, and 183.69% compared with the MC group, respectively; the HPDF group mice excreted the highest number of fecal pellets, while the HP group mice excreted the lowest, followed by the HDF group mice.

### 3.4. Effects of Dietary Intervention on the Gastrointestinal Motility Indicators of Constipated Mice

Small bowel propulsion rate is a key indicator for assessing intestinal function, which directly reflects the speed of gastrointestinal content movement through the digestive tract. Small bowel propulsion rate affects the digestion and absorption of nutrients and is closely related to intestinal health problems, such as constipation [45]. As shown in Figure 3E, the results indicated that the small intestinal propulsion rate of mice in the model group decreased significantly (*p* < 0.05) compared to the control group with an average decrease of 17.94%. As compared to the model group, the small intestinal propulsion rate significantly increased in all groups, especially in the HP (*p* < 0.01) and HPDF (*p* < 0.05) groups, showing increases of 52.72% and 38.26%, respectively. A high small intestinal propulsion rate may lead to a short retention time of food debris in the intestine and insufficient water absorption. An appropriate small intestinal transit rate helps to form soft and hard stools that are easy to pass and maintains intestinal health [46]. Su et al. [47] found that various doses of lotus seed oligosaccharides and resistant starch mixtures could significantly increase the small intestinal propulsion rate, shorten the defecation time, promote intestinal peristalsis, and prevent constipation in mice. Dietary fiber shortens the retention time of food residue in the intestines by increasing the volume of surimi and stimulating intestinal peristalsis, reduces the over-absorption of water, and prevents the feces from drying out and hardening, which in turn can have a laxative effect [48,49]. The current study results showed that all groups significantly increased the small bowel propulsion rate and improved the small bowel peristalsis. Among them, the HPDF and HDF groups more appropriately regulated the level of small intestinal peristalsis; in particular, the recovery level of the HPDF group was closer to that of the control group.

Gastric emptying rate is defined as the rate at which stomach contents enter the duodenum. It is one of the most important indicators for assessing gastric motility. The stronger the gastric motility, the faster the gastric emptying rate [50]. The effects of each group on the gastric emptying rate of constipated mice are shown in Figure 3F. As compared to the control group, the gastric emptying rate of the model group mice decreased significantly (*p* < 0.0001) by 13.26%. As compared to the model group, the gastric emptying rate increased in the HPDF (*p* < 0.01), HP, and HDF groups (*p* < 0.05), with the highest average gastric emptying rate of 63.91% in the HPDF group, followed by the gastric emptying rates of 62.28% and 60.8% in the HP and HDF groups, respectively. This relief effect of the HP and HPDF groups was closer to that of the control group. The results showed that the HP, HPDF, and HDF groups could improve gastric emptying rates and enhance gastrointestinal motility.

### 3.5. Effect of Dietary Intervention on the Intestinal Fluorescence Image and Histopathology of Constipated Mice

**Figure 4:**
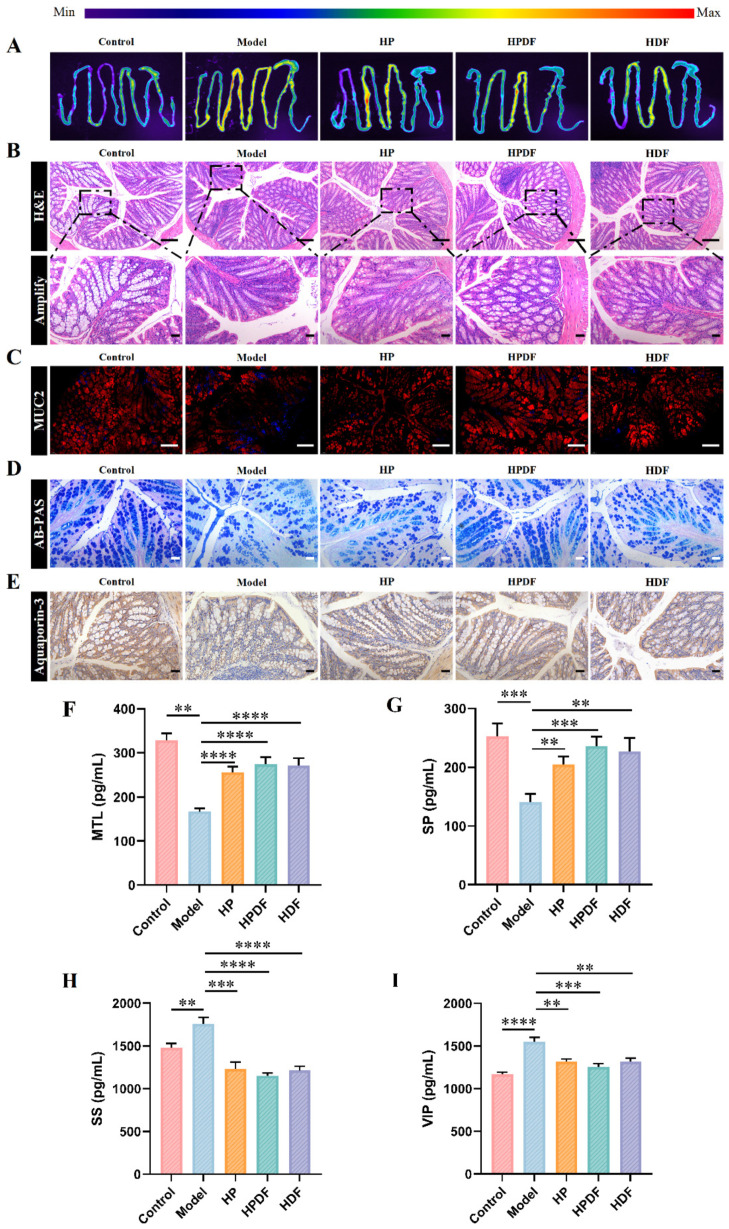
Effect of dietary intervention on the histopathological and intestinal neurotransmitters of constipated mice. (**A**) Intestinal fluorescence image. (**B**) H&E-staining, Scale bar: 100 μm for above images and 10 μm for below images. (**C**) MUC2 immunofluorescence staining, Scale bar: 100 μm. (**D**) AB-PAS staining, Scale bar: 10 μm. (**E**) Aquaprin-3 staining, Scale bar: 10 μm. (**F**–**I**) Quantitative analysis of intestinal neurotransmitters (motilin (MTL), substance P (SP), somatostatin (SS), and vasoactive intestinal peptide (VIP)) in blood. Data were represented as mean ± SD (*n* = 6). ** *p* < 0.01, *** *p* < 0.001, **** *p* < 0.0001. HP: high protein diet; HPDF: high protein and dietary fiber diet; HDF: high dietary fiber diet.

The Small Animal In Vivo Imaging tool is an advanced technological device for real-time in vivo observation of biological processes in small animals. It combines optical, nuclear medicine, or magnetic resonance imaging (MRI) techniques to achieve dynamic monitoring at the molecular, cellular, and tissue levels in small animals [34]. In this study, a small animal live imager was used to observe the chlorophyll contents in the intestines of mice, indirectly reflecting the blockage in the constipated mice intestines. As shown in Figure 4A, more red signals in the fluorescence imaging map indicated more chlorophyll content, suggesting more feces in the intestines and severe intestinal blockage. As compared to the control group, the intestinal fluorescence imaging of the model group showed a significantly more red continuous color, indicating severe blockage of the mice’s intestinal tract with no discharge of feces for a long time. As compared to the model group, the signal intensity of the HP, HPDF, and HDF groups decreased significantly, especially that of the HPDF and HDF groups, which had almost no red signal intensity. The results indicated that the HPDF and HDF groups could more intuitively alleviate the problem of intestinal blockage in constipated mice.

The results of H&E staining of mouse tissue sections are shown in Figure 4B. The cup cells and crypts in the colon of the control group mice were undamaged with intact villi. As compared to the control group, the model group mice had significantly fewer epithelial cells and cup cells, shorter and more disorganized villi, thinner muscle thickness, and impaired crypt expansion. Constipation, on the one hand, increases the fecal retention time in the colon, leading to excessive absorption of water as well as dry and hard feces, which may cause mechanical damage to the intestinal wall as it passes through the colon. The retained feces may irritate the intestinal wall, triggering an inflammatory response [51]. Chronic inflammation and mechanical injury may destroy the connective tissue of the colon. Moreover, constipation can damage the colonic epithelium, disrupting the intestinal mucosal barrier, which in turn affects colonic transit function [52]. The dietary intervention in each group alleviated the thickness of the mouse colon muscle layer and damage to the cup cells and crypts to different degrees. The improvement effects were more obvious in the HPDF and HDF groups compared to the HP group; the HPDF group had a thicker upper layer of muscle, longer crypt cells, a more intact villus structure, and more cup cells and epithelial cells.

As shown in Figure 4C, the tight junction protein Muc2, mainly secreted by cup cells [53], was probed using immunofluorescence in the colon tissues of various mice groups. As compared to the control group, the signal intensity of Muc2 protein decreased significantly in the model group, while those in the HP, HPDF, and HDF groups gradually recovered, with the best recovery in the HPDF group.

As shown in Figure 4D, mucus secretion was analyzed using AB-PAS staining to determine the effect of each treatment group on the mice intestinal mucosal barrier [54]. The model group mice exhibited significantly lower levels of mucus around the crypts and cup cells, showing evident damage to the mucus layer, as compared to the control group mice. The HPDF group had a more regular distribution of mucus and a more intact mucus layer than the model group. In contrast, the HP and HDF groups had relatively less mucus and an uneven distribution. Aquaporin-3-mediated transmembrane transport of water molecules indicates water–fluid metabolism in the colon, and it has a strong influence on the extent of water absorption in the colon and fecal transport in the large intestine [32,55].

As shown in Figure 4E, the detection of Aquaporin-3 expression level in the colon tissues of each mice group using immunohistochemical staining indicated that the HP and HPDF groups had significantly higher expressions than those in the model and HDF groups and similar to that in the control group. The elevated Aquaporin-3 protein expression decreases water reabsorption in the colon, alters cellular water permeability, and increases fecal water content, which in turn promotes defecation and relieves constipation [55]. In summary, the HPDF group showed the most obvious improvement with the histopathological structure, Muc2 level, mucus level, and Aquaporin-3 protein expression closer to that of the control group. This indicated that dietary fiber and protein together could more effectively alleviate the constipation of mice.

### 3.6. Effect of Dietary Intervention on the Intestinal Neurotransmitters of Constipated Mice

Damage to colonic tissues may affect the release of neurotransmitters in the enteric nervous system, leading to decreased peristaltic function. The current study focused on investigating the alleviating effects of different diets on constipated mice by studying the changes in excitatory (SP and MTL) and inhibitory (SS and VIP) gastrointestinal peptides. The serum levels of SP and MTL were significantly lower, while those of SS and VIP were significantly higher in the model group as compared to the control group (Figure 4F–I). SP promotes gastric emptying and small intestinal peristalsis by facilitating the contraction of gastrointestinal smooth muscle [10]. MTL is a digestive tract hormone that promotes intestinal contractile motility and accelerates intestinal peristalsis, which in turn relieves constipation [56]. As compared to the model group, the serum levels of SP (*p* < 0.01) and MTL (*p* < 0.0001) increased significantly in the HP, HPDF, and HDF group mice, with levels peaking in the HPDF group at 236.01 and 273.83, respectively. This indicates that all three diets increased excitatory gastrointestinal peptides in mice. Meanwhile, the serum levels of SS and VIP in all the groups decreased significantly, probably due to the decrease in inhibitory gastrointestinal peptides, which increased the release of neurotransmitters and thus promoted intestinal peristalsis [57]. These findings align with previous results of Sun et al. [58]. In summary, HP, HPDF, and HDF affected the release of intestinal neurotransmitters in mice, with HPDF showing the most significant improvement.

### 3.7. Effect of Dietary Intervention on Diversity of Gut Microbiota in Constipated Mice

**Figure 5:**
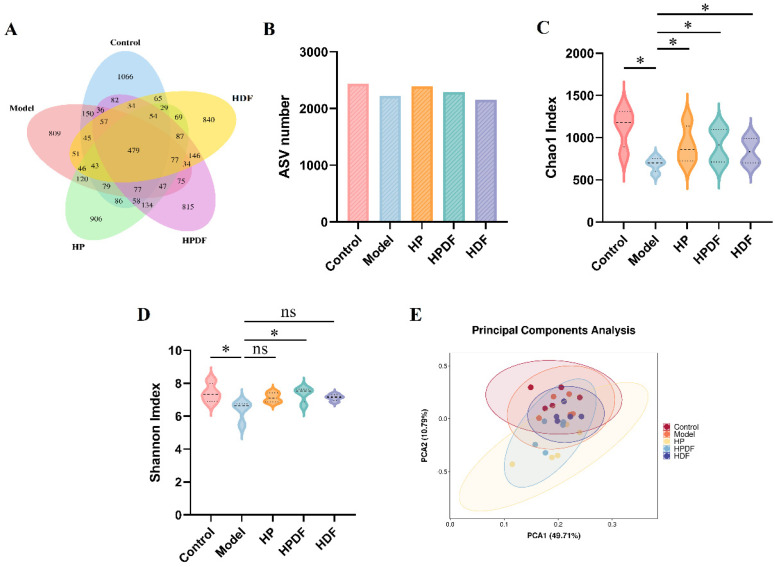
Effect of dietary intervention on the gut microbial diversity of constipated mice. (**A**) Venn diagram summary of the numbers of ASV. (**B**) ASV number. (**C**) Chao1 indices. (**D**) Shannon indices. (**E**) PCA score plots. ns indicates not significant, * *p* < 0.05. HP: high protein diet; HPDF: high protein and dietary fiber diet; HDF: high dietary fiber diet.

Amplicon sequence variant (ASV) is a feature sequence obtained by noise reduction of valid data using DADA2 and is equivalent to clustering at 100% similarity, greatly improving the species resolution circle while reducing the proportion of false positives [59]. The ASV indices of each group are shown in Figure 5A,B. The control group had an ASV index of 2440 for gut microbiota with 1066 unique ASVs, and the model group had an ASV index of 2225 with 809 unique ASVs. The HP, HPDF, and HDF groups had ASV indices of 2391, 2292, and 2156 with 906, 815, and 840 unique ASVs, respectively. As compared to the model, the ASV index and specificity increased in both the HP and HPDF groups, suggesting that dietary fiber and protein could enhance the species abundance of the constipated mice gut microbiota to some extent.

Alpha-diversity is mainly used to reflect the richness and evenness of species. Figure 5C,D show the Shannon and Chao1 indices in α-diversity. The Shannon index reflects the diversity of microbial communities; the larger the Shannon index, the more unknown factors in the community and the higher the diversity. The Chao1 index primarily reflects community richness, with the larger Chao1 index indicating a greater variety of species contained [60]. The dietary intervention significantly increased the Shannon and Chao1 indices in all groups (*p* < 0.05) as compared to the model, with the HPDF group showing the highest indices of 7.35 and 908.4, respectively. This indicated that HPDF effectively increased the diversity and richness of gut microbiota in constipated mice.

β-diversity refers to the variability of species between different samples, and principal component analysis (PCA) reflects the variability between species. As shown in Figure 5E, the contributions of coordinate PCA1 and PCA2 to the differences in gut microbiota were 10.79% and 49.71%, respectively. As compared to the control group, all the groups showed some deviations and partial overlaps, indicating that constipation altered the composition of gut microbiota in mice. The structure of mice gut microbiota in the HP and HPDF groups showed more deviation compared to the model group, with the HDF group completely different from the model group. This suggested that the combined effect of protein and dietary fiber greatly impacted the structure of constipated mice gut microbiota.

### 3.8. Effect of Dietary Intervention on the Abundance of Gut Microbiota at Phylum Level in Constipated Mice

**Figure 6:**
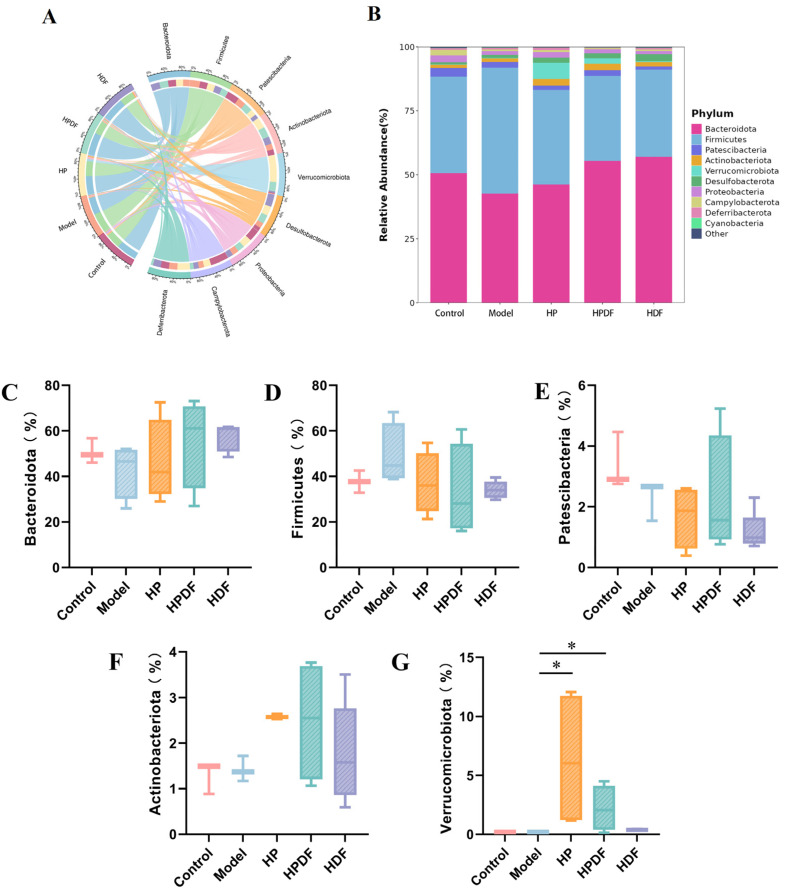
Effect of dietary intervention on the gut microbial composition of phylum level species in constipated mice. (**A**) Circos diagram of the relationship. (**B**) Phylum level distribution map. (**C**–**G**) Major microbiota. * *p* < 0.05. HP: high protein diet; HPDF: high protein and dietary fiber diet; HDF: high dietary fiber diet.

The species composition of different diets on the mice gut microbiota at the phylum level is shown in Figure 6A–G. *Bacteroidota* and *Firmicutes* are the two major phyla. As compared to the control group, the relative abundance of *Bacteroidota* in the model group decreased, while that of *Firmicutes* tended to increase but not significantly (Figure 6C,D). Shi et al. [61] found a significant increase in the relative abundance of *Firmicutes* in the intestines of constipated mice, along with an increasing trend in the relative abundance of *Bacteroidota*, which was reversed by treatment with a complex fruit drink. In the current study, the relative abundances of *Bacteroidota* and *Verrucomicrobiota* increased in each group after dietary intervention, while that of *Firmicutes* decreased. Zhang et al. [10] observed a decrease in *Bacteroidetes* and an increase in *Firmicutes* in the model group, which was reversed by COS treatment. We observed a similar trend in our study. Among them, the intervention effect of the HPDF group was the most obvious. *Bacteroidota* can absorb degraded polysaccharides and proteins, improve nutrient utilization, accelerate the formation of intestinal mucosal blood vessels, and maintain the intestinal microecological balance [62]. The presence of *Verrucomicrobiota* is beneficial in restoring secondary bile acid levels in the mouse intestine [63]. Wang et al. [64] showed that *Verrucomicrobia* was positively correlated with bile acid and deoxycholic acid content at the phylum level, and gut microbiota could break down saffron dietary fiber into short-chain fatty acids, thereby improving the composition of the gut microbiota.

### 3.9. Effect of Dietary Intervention on the Abundance of Gut Microbiota at Genus Level in Constipated Mice

**Figure 7:**
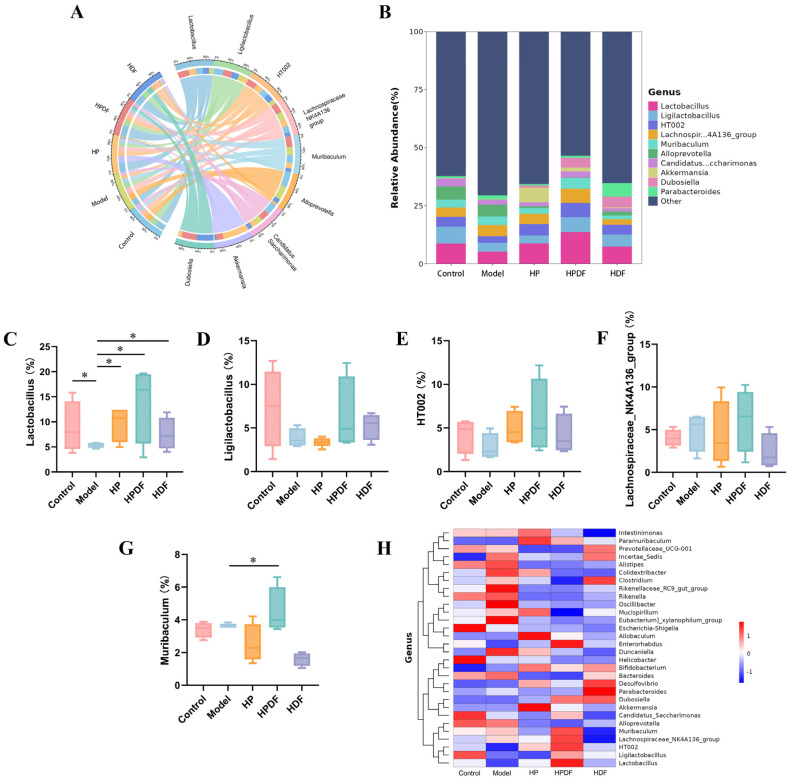
Effect of dietary intervention on the gut microbial composition of genus level species in constipated mice. (**A**) Circos diagram of the relationship. (**B**) Genus level distribution map. (**C**–**G**) Major microbiota. (**H**) Heat map analysis of relative abundance. * *p* < 0.05. HP: high protein diet; HPDF: high protein and dietary fiber diet; HDF: high dietary fiber diet.

The effects of dietary intervention on the level of gut microbial genera in constipated mice were further investigated as shown in Figure 7A–G. *Lactobacillus* and *Ligilactobacillus* are the dominant genera, which maintain the balance of gut microbiota, inhibit the growth of pathogenic bacteria and pro-inflammatory factors, enhance the function of the intestinal barrier, reduce intestinal permeability, and prevent intestinal infection and inflammation [65,66]. The abundances of *Lactobacillus* and *Ligilactobacillus* were significantly lower (*p* < 0.05) in the model group as compared to the control group, and their abundances were higher in the HPDF intervention compared to those in the model and control groups. Lan et al. [3] found a significant reduction in the abundance of *Lactobacillus* in a constipation mouse model induced by compound diphenoxylate, and inulin intervention elevated the contents of *Lactobacillus*. In addition, a heat map analysis of the relative abundance of the top 30 gut microbial genera was performed to assess the distribution of gut microbes in each group of mice (Figure 7H). *Lactobacillus* and *Ligilactobacillus* were the two most abundant genera. The abundance of beneficial bacteria, such as *Lactobacillus* and *Ligilactobacillus*, increased significantly in the HPDF group after dietary modification. In summary, HPDF regulates the imbalance of the gut microbiota in constipated mice by promoting the growth of beneficial bacteria. This led to improving the intestinal microecological environment and regulating the stability of the intestinal environment to alleviate constipation.

## 4. Conclusions

The optimal formulation of AKSG, containing 5.5% inulin, 1% ADSP, 1% EWP, 0.9% SA, and 0.4% lys, was determined by single factor and response surface tests. The effects of HP, HPDF, and HDF on loperamide hydrochloride-induced constipation in mice were analyzed based on feces-related indicators, gastrointestinal motility, intestinal neurotransmitters, and gut microbiota. The results showed that all the interventions could promote intestinal peristalsis, regulate the level of intestinal neurotransmitters, and improve the state of colonic tissues, thus relieving constipation. Among them, HPDF showed the most significant improvement, significantly increasing defecation volume, fecal water content, small intestinal propulsion rate, and gastric emptying rate in constipated mice. Moreover, HPDF increased the abundances of *Lactobacillus* and *Ligilactobacillus* and decreased that of *Firmicutes*, thus improving the composition of gut microbiota. These results suggest that dietary fiber synergized with protein is more effective in relieving loperamide hydrochloride-induced constipation. Therefore, the high dietary fiber AKSG might be a functional food for the relief of dysphagia constipation.

## Figures and Tables

**Table 1:** AKSG formulation.

Ingredients	Weight (g)
Antarctic krill	40
*Litopenaeus vannamei*	60
Ice water	15
NaCl	1
Soybean oil	0.5
Monosodium glutamate	0.5
Inulin	3
EWP	3
ADSP	3
SA	0.5
Lys	0.4

**Table 2:** Feed formulation.

Ingredients	Basic Feed (%)	HP (%)	HPDF (%)	HDF (%)
Corn	28.4	19.88	19.88	26.56
Wheat middlings	28.6	20.02	20.02	26.74
Soybean meal	16	11.2	11.2	14.96
Flour	18	12.6	12.6	16.83
Vegetable oil	1.6	1.12	1.12	1.5
Calcium bicarbonate	1.6	1.12	1.12	1.5
Fishmeal	1.5	1.05	1.05	1.4
Additive	4.3	3.01	3.01	4.02
AKSG without dietary fibre	—	30	—	—
AKSG	—	—	30	—
Inulin	—	—	—	5.5
Sodium alginate	—	—	—	1

## Data Availability

The original contributions presented in the study are included in the article; further inquiries can be directed to the corresponding author.

## Supplementary Materials

The following supporting information can be downloaded at: https://www.mdpi.com/article/10.3390/foods15010059/s1, Table S1: Response surface test factors and levels, Table S2: Design and results of response surface test, Table S3: Analysis of variance in the response surface analysis.
