# Supplementary material for: Exploring the Effects of High Protein and High Inulin Composite Shrimp Surimi Gels on Constipated Mice by Modulating Gastrointestinal Function and Gut Microbiota
Source: Foods. 2025 Dec 24;15(1):59. doi: 10.3390/foods15010059 (PMC12786157; doi:10.3390/foods15010059)
Supplement: Supplementary file 1 [file foods-15-00059-s001.zip › foods-4021182-supplementary.pdf]

Table S1 Response surface test factors and levels.

Factor	level		
	-1	0	1
A Sodium alginate (%)	0.5	0.7	0.9
B Inulin (%)	4	5	6
C Lysine (%)	0.2	0.3	0.4

Table S2 Design and results of response surface test.

Run	A Sodium alginate (%)	B Inulin (%)	C Lysine (%)	Y Hardness (g)
1	0.7	4	0.2	874.19
2	0.7	5	0.3	776.50
3	0.9	6	0.3	856.36
4	0.7	5	0.3	775.75
5	0.7	6	0.2	809.36
6	0.9	5	0.4	708.88
7	0.5	4	0.3	961.42
8	0.9	4	0.3	793.19
9	0.7	5	0.3	809.33
10	0.9	5	0.2	781.44
11	0.5	5	0.2	843.68
12	0.7	5	0.3	769.43
13	0.5	5	0.4	813.43
14	0.5	6	0.3	905.40
15	0.7	6	0.4	807.94
16	0.7	5	0.3	770.52
17	0.7	4	0.4	815.28

Table S3 Analysis of variance in the response surface analysis.

Source	Sum of squares	df	Mean square	F-value	p-value	
Model	53266.442	9	5918.494	17.104	0.0006	Significant
A-SA	18437.236	1	18437.236	53.284	0.0002	
B- Inulin	528.493	1	528.493	1.527	0.2564	
C-Lys	3327.000	1	3327.000	9.615	0.0173	
AB	3552.001	1	3552.001	10.265	0.0150	
AC	447.579	1	447.579	1.294	0.2928	
BC	826.512	1	826.512	2.389	0.1661	
A^2	3658.401	1	3658.401	10.573	0.0140	
B^2	20227.546	1	20227.546	58.458	0.0001	
C^2	2212.333	1	2212.333	6.394	0.0393	
Residual	2422.142	7	346.020			
Lack of Fit	1330.385	3	443.462	1.625	0.3177	Not significant
Pure Error	1091.757	4	272.939			
Cor Total	55688.585	16				
